# Managing Big Data for Addressing Research Questions in a Collaborative Project on Automated Driving Impact Assessment

**DOI:** 10.3390/s20236773

**Published:** 2020-11-27

**Authors:** Francesco Bellotti, Nisrine Osman, Eduardo H. Arnold, Sajjad Mozaffari, Satu Innamaa, Tyron Louw, Guilhermina Torrao, Hendrik Weber, Johannes Hiller, Alessandro De Gloria, Mehrdad Dianati, Riccardo Berta

**Affiliations:** 1 Department of Electrical, Electronics and Telecommunication Engineering and Naval Architecture (DITEN), University of Genova, 16145 Genova, Italy; Nisrine.Osman@elios.unige.it (N.O.); alessandro.degloria@unige.it (A.D.G.); riccardo.berta@unige.it (R.B.); 2 Warwick Manufacturing Group (WMG), University of Warwick, Coventry CV4 7AL, UK; E.Arnold@warwick.ac.uk (E.H.A.); Sajjad.Mozaffari@warwick.ac.uk (S.M.); M.Dianati@warwick.ac.uk (M.D.); 3 VTT Technical Research Centre of Finland Ltd., P.O. Box 1000, FI-02044 VTT Espoo, Finland; Satu.Innamaa@vtt.fi (S.I.); Guilhermina.Torrao@vtt.fi (G.T.); 4 Institute for Transport Studies, University Road, University of Leeds, Leeds LS2 9JT, UK; T.L.Louw@leeds.ac.uk; 5 Institute for Automotive Engineering, RWTH Aachen University, Steinbachstr 7, 52074 Aachen, Germany; hendrik.weber@ika.rwth-aachen.de (H.W.); johannes.hiller@ika.rwth-aachen.de (J.H.)

**Keywords:** research data collection and sharing, connected and automated driving, deployment and field testing, vehicular sensors, impact assessment, knowledge management, collaborative project methodology

## Abstract

While extracting meaningful information from big data is getting relevance, literature lacks information on how to handle sensitive data by different project partners in order to collectively answer research questions (RQs), especially on impact assessment of new automated driving technologies. This paper presents the application of an established reference piloting methodology and the consequent development of a coherent, robust workflow. Key challenges include ensuring methodological soundness and data validity while protecting partners’ intellectual property. The authors draw on their experiences in a 34-partner project aimed at assessing the impact of advanced automated driving functions, across 10 European countries. In the first step of the workflow, we captured the quantitative requirements of each RQ in terms of the relevant data needed from the tests. Most of the data come from vehicular sensors, but subjective data from questionnaires are processed as well. Next, we set up a data management process involving several partners (vehicle manufacturers, research institutions, suppliers and developers), with different perspectives and requirements. Finally, we deployed the system so that it is fully integrated within the project big data toolchain and usable by all the partners. Based on our experience, we highlight the importance of the reference methodology to theoretically inform and coherently manage all the steps of the project and the need for effective and efficient tools, in order to support the everyday work of all the involved research teams, from vehicle manufacturers to data analysts.

## 1. Introduction

Solving grand challenges, such as automated connected driving, often requires collaboration across multiple domains and technical areas. A key factor in the success of collaborative research projects is the methodology and the relevant tools used to address the challenges. Some collaborative tools are well established (e.g., for managing the project and its risks, sharing documentation and source code, etc.), while others are less general and more related to application-specific tasks. The literature is rich in guidelines, techniques and tools for general project challenges (e.g., [1,2]), but there is a lack of specific information and tools for different partners to deal with sensitive data in order to answer a set of research questions (RQs) at project level. This task is gaining relevance in the current industrial research context, where there is a growing focus on big data, especially from ever more pervasive and sophisticated sensors and on extracting meaningful information from them. However, while there is significant work published on application-oriented data analysis (e.g., [3]), we found a lack in data management for assessing the impact of the new technologies’ adoption.

We thus intend to investigate how to organize a robust workflow for quantitatively addressing RQs in a collaborative project sharing sensitive data among various partners, while ensuring methodological soundness and data validity and protecting partners’ intellectual property (IP).

We think that the automated driving sector represents a highly significant investigation domain given the huge amount of research that is being carried out in the field (e.g., [4,5,6]). As an example use case, we thus discuss our experience in a 34-partner EU-funded project, L3Pilot, which is assessing the impact of Society of Automotive Engineers (SAE) Level 3 (L3) and Level 4 (L4) automated driving functions (ADFs). Tests are being conducted in pilots in 10 European countries, with vehicles provided by 13 vehicle owners (original equipment manufacturer (OEM), suppliers or research facilities).

The L3Pilot RQs cover different leading-edge ICT adoption impact assessment areas, including (i) technical performance of the tested L3 ADFs, (ii) user acceptance and behaviour, (iii) impact on traffic and mobility and (iv) societal impacts (see L3Pilot Deliverable D3.1 [7]). This paper presents how we implemented a reference methodology for large scale pilots and field operational automotive tests—namely Field opErational teSt supporT Action (FESTA) [8]—in order to get the quantitative information needed to answer the project’s research questions (RQs). A key novelty in this process is the use of the Consolidated Database (CDB), which allows data from all the pilot sites to be shared anonymously and securely amongst project partners to facilitate data analysis aimed at answering the project’s RQs.

This paper presents the challenges we have faced in implementing the methodology and consequently developing a coherent, robust workflow. First, we needed to quantitatively capture each RQ’s requirements in terms of raw data to be collected during the tests, so as to allow a proper investigation. Then, we set up the needed tools in an iterative development process involving several partners (vehicle manufacturers, research institutions, suppliers and developers), with different perspectives and requirements. Finally, we deployed the system so that it is fully integrated within the project’s data toolchain and usable by all the partners.

It is important to highlight that this paper focuses on the method, workflow and tools and does not discuss the actual domain-specific data, which will be the subject of another publication.

The remainder of the paper is organized as follows. Section 2 gives an overview of the related work, while Section 3 presents the methodology and the consequent specifications for the target process. Section 4 presents the design and implementation of the CDB. Section 5 discusses what we have learnt from the deployment of the system, while Section 6 draws the final conclusions.

## 2. Related Work

Beside the general overviews cited in the Introduction, there is a rich literature on privacy and risk management in projects. For instance, [9] deal with Risk Assessment in Multi-Disciplinary Engineering Projects, [10] with privacy risks when sharing data on information systems. Furthermore, [11] investigates the validity of sharing privacy-preserving versions of datasets. They propose a Privacy-preserving Federated Data Sharing (PFDS) protocol that each agent can run locally to produce a privacy-preserving version of its original dataset. The PFDS protocol is evaluated on several standard prediction tasks and experimental results demonstrate the potential of sharing privacy-preserving datasets to produce accurate predictors. In addition, [12] provides an extensive review of data analytic applications in road traffic safety, with particular attention to crash risk modelling.

Furthermore, [13] deals with integrating diverse knowledge through boundary spanning processes, with a particular focus on multidisciplinary project teams. The concept of a Project Consortia Knowledge Base (PC-KB) is presented in [14] in an integration framework based on semantic knowledge that facilitates project-level communication as well as access to project data across tool and partner boundaries.

Commercial companies (e.g., Amazon, Microsoft, Google) have established efficient cloud ecosystems for data management providing very powerful services, but they rely on proprietary technologies, with very limited interoperability and development opportunities for third parties. However, we could not find in the literature guidelines on how to exploit these cloud technologies to support project partners in processing big data to address quantitative research questions.

In recent years, a number of field operational tests (FOTs) have been executed to test new Advanced driver-assistance systems (ADAS) in authentic traffic conditions, involving thousands of drivers (e.g., euroFOT [15,16]). With a view to ensure scientific soundness, the Field opErational teSt supporT Action (FESTA) project developed a methodology for field operational tests (FOTs), with three main focuses: user, vehicle, context [8]. This methodology is described in the FESTA Handbook which has been frequently updated according to the latest lessons learned [17]. It records lessons learned and provides best practices collected in several European FOTs in the last ten years. L3Pilot decided to adopt this methodology, as illustrated in the next section.

Several collaborative industrial research projects have been conducted in Europe addressing the first levels of automated driving. The AdaptIVe project developed several functionalities providing various levels of driver assistance, such as partial, conditional and high automation [18]. Drive C2X investigated cooperative awareness, which was enabled by periodic message exchange between vehicles and roadside infrastructure [19,20]. The FOT-Net Data project prepared the Data Sharing Framework, which provides hands-on recommendations on how to manage data sharing of data from the transportation research area [21]. The TEAM project developed an app suite for supporting collaborative road mobility [22].

## 3. Methodology

### 3.1. Overview

The RQs for all impact areas in the L3Pilot project (listed in the Introduction, see also [7]) were generated through the top-down approach recommended by the FESTA Handbook [17]. The process began with a review of the descriptions of automated driving functions (ADFs) that were going to be piloted during the project. Therefore, in the early stages, only high-level RQs (Levels 1 and 2 in Table 1 example) were defined, to meet the project objectives.

In such a top-down approach, the generation RQs and hypotheses typically is based on theoretical understanding of the mechanisms how different impact areas are influenced. Thus, the process was started with an extensive literature review, which aimed to identify the key elements related to different impact areas. In addition, the review aimed to find knowledge gaps. The process was iterative (generate RQs, review them) to ensure that all major topics were covered and RQs were well formulated. In this step, a wide range of RQs was created, not limiting them by means of any single data collection method. The RQs were simply based on literature and the experience of the project members in previous, related work. The generation of the first (higher) level of research questions was structured according to the four L3Pilot evaluation areas. The second stage involved the development of more detailed RQs related to specific components of the higher-level questions, where appropriate. For each RQ, the underlying hypothesis is then made explicit. Table 1 provides an example in the Technical and Traffic area.

The top-down approach to setting RQs was followed by a bottom-up revision. In that phase, the RQs were cross-checked for their feasibility in terms of the data generation, a suitable experimental procedure at the pilot sites and availability of evaluation methods and tools [23]. RQs were prioritized based on test site characteristics, data coding and processing demand, ethical constraints, resources and time available in the project and importance for the research. In this phase, some of the first two levels of RQs were updated to be in line with the evaluation possibilities in the project.

**Figure 1:**
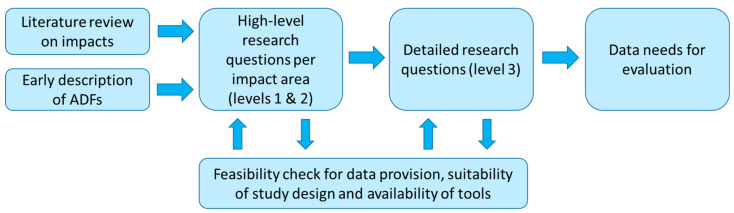
Overview of the research questions (RQ) definition and implementation workflow.

In line with the FESTA Handbook, the next steps after generation of the hypotheses concerned the definition of the relevant performance indicators (we cover them in detail in the next subsections) and of the logging needs related to them. Here, we differentiated the subjective and objective data [7]. Questionnaires would collect subjective data across test participants (drivers and possible passengers), and objective data would be collected mostly from the data loggers of the test vehicles, additional cameras installed on them, and, when necessary, from external data sources (e.g., weather information, road type, etc.) [24]. Figure 1 provides an overview of the RQ definition and implementation workflow.

### 3.2. Objective (Vehicular) Data

The adopted methodology requires defining (i) the performance indicators (PIs) through which the RQs can be answered, (ii) the derived measures that are needed to calculate these indicators, and (iii) the actual vehicular signals that are needed to calculate these measures (see the full process in [24]). Beside the standard vehicular signals (e.g., speed, acceleration, pedal activity, etc.), source data come also from state-of-the-art automated driving sensors, such as cameras, Light Detection and Ranging (lidars) and radars. As a single PI may be derived from different alternative derived measures—and a single measure from different alternative signals—a collaboration was set up among the evaluation team (who express the data needs), the vehicle owners (who provide and share the data) and those responsible for developing the processing tools. The collaboration aimed at defining performance indicators (PIs) and derived measures for all RQs [7], as illustrated in the Table 1 example. The overall analysis defined a set of signals to be provided by all the vehicle owners that are specified in a common data format [25].

Four different types of PIs (Table 2) were defined to be computed from the vehicular signals and stored in the Consolidated database (CDB) as the factual basis for answering the RQs. PIs are typically constituted by statistical aggregations (e.g., avg/std/min/max) in significant intervals of a trip. Two PI types are computed at trip level: while Trip_PIs are general indicators synthetizing a trip, ScenarioSpecific_TripPIs are computed aggregating trip segments from a specific scenario only. The other two PI types (namely, ScenarioInstance_PI and Datapoints) are much more specific, as they are computed for each instance of a given driving scenario detected during a trip. Table 2 provides an overview of the L3Pilot vehicular PI types, with some examples. The table reports only two Datapoint types, as examples, since there is one datapoint type for each driving scenario type.

Several research questions required analysing context data beyond the actual vehicular signals and questionnaire answers. Context data were useful, particularly to segment information so to allow comparisons and more focused analysis. Among context data we highlight:

- Experimental conditions. Different conditions have to be considered, such as: baseline, ADF not available, ADF off, ADF on.
- Road types. Tests are performed on various road types, such as: motorways, major urban arterials, other urban roads.
- Driving scenarios. The system has to track different types of driving scenarios, that are typical driving situations, such as uninfluenced driving, lane change, lane merge, following a lead vehicle, etc. Scenarios are computed by the L3Pilot data toolchain, processing the vehicular time series [26].

So, Trip PIs are to be computed for different segments, based on the actual experimental condition (i.e., baseline, ADF off, ADF on) and road type; and Scenario Instance PIs are to be segmented not only on the basis of the scenario type itself, as per definition, but also considering the different experimental conditions and road types.

Other metadata were mandated as well, such as driver type (professional or ordinary), temperature and speed limits, in order to better characterize the context of each PI measurement.

### 3.3. Confidentiality

There are several external factors that impact the extent to which the project RQs can be addressed. For example, when assessing the effects of the ADFs on driving (i.e., observed differences between the ADF and human driver with respect to, e.g., car-following behaviour), the maturity of the system is important to take into account and whether the test vehicle represents the driving experience of a mature product. In addition, to get the open road testing permission for an ADF, public authorities demand safety of testing. The details of the driving dynamics can also be a sensitive topic to the manufacturers. For that reason, it is important to make sure that vehicle telemetry data are kept confidential, and that the manufacturers or their tested systems cannot be identified, ranked or compared from any information shared within the project or with others, as their competitiveness at a crucial development stage would be compromised. Another challenge for gaining a good understanding of the impacts of ADFs is that the data is limited to specific test routes, speed ranges and weather conditions, which are defined by the function’s Operational Design Domains (ODD). All pilot studies must also adhere to the rules of respective OEM and to national regulations on testing of automated driving, like the mandatory use of safety drivers in certain situations. Therefore, all this limits the possibilities of the project to address all of the RQs set for evaluation. To address these limitations, L3Pilot has specified a common methodology, data format and analysis toolkit, which will be used across all pilot sites and for evaluation of different ADFs [26]. Other solutions include the use of a dedicated research partner for analysis of data from a single pilot site to ensure that the access to commercially sensitive data is controlled and that data is pseudonymised before uploading to the CDB and shared among partners.

The CDB includes aggregated data from several sites in such a manner that commercially sensitive information is protected. A key requirement for being able to merge data is, that, in addition to protecting the privacy of manufacturers, the result is meaningful for the user of the result as such or those performing the following evaluation steps. In other words, the results must describe the impacts of automated driving but without compromising this privacy. The merging of data from different sites also leads to the outcome where the L3Pilot results do not represent the impact of single (OEM)-specific ADFs but the generic impacts that can be expected once these systems are introduced to the road [27].

As confidentiality is a key requirement when sharing valuable data, three main constraints were applied to data uploaded to the CDB:

- It should not be possible to identify which pilot site the data came from. For example, attention was paid not to insert metadata, such as temperature and date, that might hint to identify the location of the pilot site.
- No personal data about the driver, passengers nor other test participants.
- No possibility to characterize in detail the behaviour of ADFs. This was achieved by the fact that vehicular sensor data are not uploaded to the CDB as time series but as summarised performance indicators, which are described later.

As an ID of the trip and of the user was considered necessary to allow data owners to track their data in the CDB (also to update or delete them, if needed), an SHA-256 hashing-based pseudonymization was implemented [26]. Knowing the encrypted IDs, the data owners can track their data in the CDB, while such IDs are not decipherable by the other users.

Despite these constraints, there is no 100% guarantee that data cannot be linked to a pilot site, if malicious and highly sophisticated techniques we did not foresee are applied to the data.

### 3.4. Subjective Data

While until now we have focused on vehicular sensor data, a complete assessment of ADF functions also requires processing subjective data. One of the primary sources of data for the user and acceptance evaluation within L3Pilot is a pilot site questionnaire, which gathers subjective data from participants at the various pilot sites (for the full questionnaire see [23]). L3Pilot tests four different types of Automated Driving Functions (ADFs), including motorway, traffic jam, urban and parking. We developed a base questionnaire for background information, including questions related to sociodemographic factors, vehicle use and purchasing decisions, driving history, in-vehicle system usage, activities while driving, trip choices and mobility patterns. The data collected in the first part will be used to create different user groups for the user and acceptance evaluation. We also included questions specific to the ADF. For example, these questions assess various aspects of participants’ initial reactions to using the particular ADF. To understand whether having daily access to the ADF might change any decisions or behaviours, they were reasked questions about vehicle use and purchasing decisions, driving history, in-vehicle system usage, engagement with nondriving tasks, trip choices and mobility patterns.

### 3.5. Workflow Requirements

From a process viewpoint, requirements included efficiency of workflow, and in particular, upload and download of data to/from the CDB. We highlight in particular:

- Recursive upload. The user should define the source directory and the system should automatically detect the files to be processed and do the upload of their contents to the CDB. All the subdirectories should be recursively explored.
- CSV download. The system should allow the possibility of downloading contents in either .json or .csv format, which is the typical input format for the statistical processing packages used by the analysts. The granularity of download is at the feature level. That is, a user should be able to download all the measurements of all the accessible features (Trip_PI, Scenario_Instances, Datapoints, etc.) or only some of them.
- Postediting of the performance indicators. Once the performance indicators for a trip are computed, the data provider should be able to check and edit them before the upload to the CDB.

The above requirements were typically defined during the development, in an iterative fashion, as the data providers and analysts suggested improvements based on their working experience.

## 4. Design and Implementation of the Consolidated Database (CDB)

**Figure 2:**
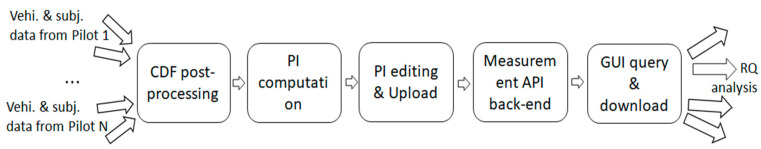
L3Pilot data workflow.

In order to meet the above presented methodological goals, L3Pilot defined a data flowchart (Figure 2), which is the basis for the system architecture. The workflow starts with data collection at the pilot sites and ends with data analysis by impact experts. At the beginning there is a different processing for vehicular sensor data (Section 4.1) and subjective data (Section 4.2), then these data are managed seamlessly. The first, fundamental step consists in translating all the heterogeneous data sources in the Common Data Format (CDF), which has been described in [25] and made publicly available [28]. The CDF postprocessing phase is described in detail in [26], while this paper focuses on the CDB. In the CDF postprocessing, the project’s analysis partners use the Derived Measures—Performance Indicator Framework (DM-PI-Framework) to enrich the vehicular signal time series from a vehicle’s trip with the computed derived measures (DM) and the detected driving scenarios, which are fundamental for the computation of CDB PIs, as described in the next subsection.

### 4.1. CDB PI Computation for Vehicular Sensor Data

The CDB PI computation step consists in synthesizing the vehicular time series so that the CDB stores only high-level information that allows tackling the project RQs, without compromising the confidentiality of the single-vehicle owner companies. This stage is undertaken by the CDB Aggregator module, which processes HDF5 files (one per each trip), containing the original time series formatted in CDF and enriched by the DM-PI-Framework’s, as mentioned above. The output of the CDB Aggregator module is represented by a set of .json files storing the computed PIs. Processing an input HDF5 file, the Aggregator produces one .json file for each one of the four PI types defined in Table 3 (i.e., Trip PI, Scenario Instance PI, etc.). The .json files are ready to be uploaded to the CDB, for instance through a well-established Application Programming Interface (API) client such as Postman, or, better, through the Uploader, a dedicated module described in Section 4.3. The same information contained in the .json files is also saved in corresponding .csv files, that are more easily readable by the analysts.

**Figure 3:**
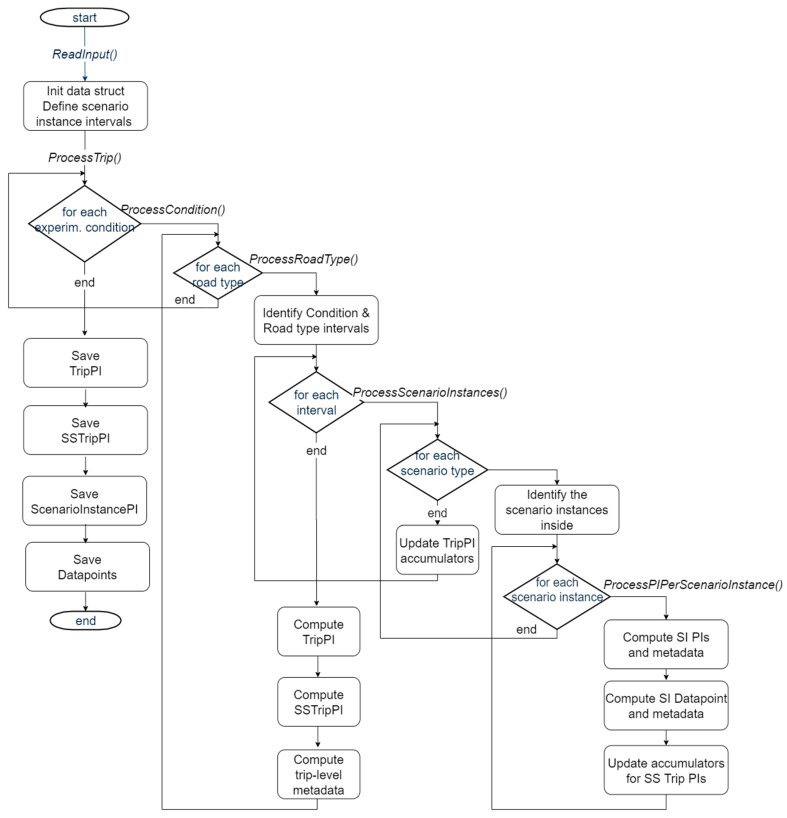
Flowchart of the vehicular sensor data processing Matlab scripts.

The CDB Aggregator module consists of a set of Matlab scripts. Figure 3 provides a high-level outlook of the programme, with three main phases: initialization, reading signals from the input HDF5 file; processing loop; and a final saving of the four types of PIs. The processing loop is the core of the programme, as it processes the time series and segments the computation of the PIs according to the context information presented in Section 3.2. First, the experimental condition is considered. Then, for each identified segment, the road type is considered. This level of segmentation leads to the computation of Trip PIs. Computation of Scenario Instance PIs and Datapoints requires further segmentation of the timeline based on the detected driving scenarios. Scenario Specific Trip PIs introduce the need for accumulating the indicator values across all the scenario instances in the trip. Similarly, the length of each scenario instance is needed for the Trip PI indicator reporting the percentage of time passed in each scenario within the trip.

**Figure 4:**
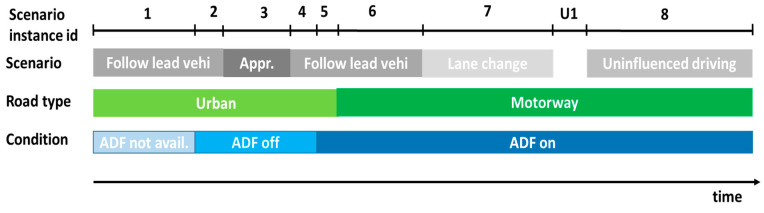
Example of scenario segmentation during a trip.

An example of the resulting segmentation is reported in Figure 4, where we can see eight different scenario instance PIs computed. A slice, indicated as Unrecognized 1 (U1), will not produce Scenario Instance PI, nor Datapoints, nor Scenario Specific Trip PI, as a scenario could not be detected there. However, the signal values contained in that segment will contribute to the Trip PI indicators in the ADF on condition.

### 4.2. Subjective Data Processing

When conducting studies across multiple sites, it is essential that any data collection methodologies are applied uniformly. For example, the pilot site questionnaires are administered across all pilot sites, which vary in many respects (e.g., country language), but most relevant here is the interexperimenter variability. Therefore, to control for this variability, the questionnaire was implemented using the online tool LimeSurvey, where the only task for the pilot site staff was to transfer the translated versions of the questionnaire into the online LimeSurvey platform, enabling the administration of the questionnaire via laptops or mobile tablet computers. The use of LimeSurvey also ensures that the data output aligns with the CDF used by a consortium-wide consolidated database.

The imported surveys may then be customised and translated versions can be implemented accordingly. Pilot sites are then able to export their newly generated surveys and/or to export their results to CSV or SPSS format (Statistical Package for the Social Sciences). Note: the SPSS output of the reference version of the questionnaire is in line with the common data format requirements for the consolidated database. Although selected partners/pilot sites responsible can create, edit or view a survey, it is imperative that the questionnaire item codes are not changed, as this is the mechanics which allows tracking responses from different pilot sites. Thus, to ensure that a CDF was applied across pilot sites, instructions on the questionnaire implementation, administration and metadata were defined at the consortium level.

In terms of the administration of the questionnaire, there are differences between pilot sites regarding the length and number of drives by each participant. Therefore, the project recommendation is that the questionnaire should be completed after the last test ride, irrespective of whether a driver has multiple drives (see further details in [29]).

**Figure 5:**
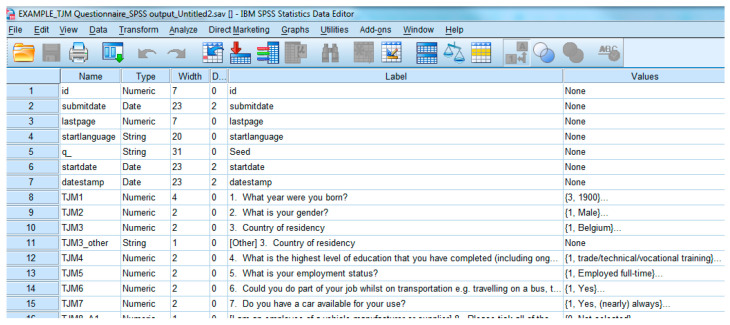
Example data output in SPSS.

Following the completion of all questionnaires, each pilot site must export the test participants’ response results to the SPSS file format (as illustrated in Figure 5), to ensure a common data format in the CDB for the user and acceptance evaluation area.

Quality checking has been implemented both for vehicular and subjective data. In this paper, we briefly present the procedure we set up for this second type of data.

Using the common LimeSurvey implementation should ensure that all questionnaire items and responses follow the nomenclature set out in the CDF for the official L3Pilot questionnaire. However, some partners used other implementations, and there is always the chance that some item names and codes deviate from the original. A data format map is prepared for each type of questionnaire, with all expected questions and possible answer codes and ranges. Before being uploaded to the CDB, a Matlab data quality script parses each questionnaire outcome file, searching for inconsistencies compared to the CDF.

A strategy is also adopted for missing data. Data may be missing because there was an error in data collection or during the data transfer process between data collection, LimeSurvey and SPSS. In order for analysts to identify cases where data is known to be missing, pilot sites are asked to fill these cells with a dummy response (1).

Should a pilot site attempt to upload a dataset including empty cells or errors from the Matlab data quality script, they will receive an error message and be asked to verify the contents of the missing cells. These steps ensure the reliability and validity of the uploaded data.

### 4.3. Uploader

The previous two steps prepare the files with the vehicular or subjective PIs to be uploaded to the CDB, via its RESTful APIs, that will be described later. For this step, a third party API development tool such as Postman can be used. However, we decided to develop an ad-hoc tool, namely the Uploader, to enhance usability, according to the specifications. This solution for uploading was preferred to a browser-based one, as it allows full access to the local file system. Given the typical On-Line Analytical Processing (OLAP) [30] pattern of usage foreseen for the CDB, we were in fact required to support an efficient upload of batches of files. The Uploader allows the user to indicate a source directory and then recursively searches in all the subdirectories all the matching .json files. Compliance is given by the name of the file, which must start with an eight-digit and end with a code indicating the type of the PI (e.g., Trip PI, Datapoint, Urban questionnaire).

Another functionality implemented by the Uploader concerns the support of postediting the csv files output by the CDB-aggregator. Postediting of PIs can be made by analysts on .csv files and the uploader offers the functionality of transforming .csv files into uploadable .json files. In addition, in this case, recursive processing of a root directory is implemented.

The Uploader is a NodeJS Command Line Interface (CLI). The user can execute some simple .json encoded command files that specify the operation to be performed on the CDB (upload, download, update, delete) or on local data (.csv to .json functionality) and the value of the corresponding parameters (e.g., source/destination directory, data override option, etc.).

### 4.4. Measurement API Back-End

For the data storage, we used Measurify (formerly, Atmosphere), an open-source RESTful API dedicated to storing measurements, typically but not exclusively from the Internet of Things [31,32]. Measurify is implemented in NodeJS and relies on MongoDB, a state-of-the-art nonrelational database, as the underlying database management system.

Measurify is a generic measurement API, which can be configured for different applications in different installations. This is achieved by inserting values for some fundamental resource collections. The most important one is the characterization of the features that describe the data type of the measurements to be uploaded and managed. This resource is used for the data integrity check at each measurement upload. For the L3Pilot installation (i.e., the CDB), we created one feature for each vehicular and subjective data type. Thus, we have four vehicular features: Trip PIs, Scenario Instance Trip PI, Datapoint (actually, since the structure of a Datapoint is scenario-dependent, we have one feature for each driving scenario type—Table 2), and Scenario Instance PI; and three subjective features: urban questionnaire, motorway questionnaire and parking questionnaire. In order to allow such a conceptually straightforward mapping, the Measurify’s data structure needed to be extended so to contain data of different dimensions in each item. For instance, a measurement with a feature of type Trip PI has some scalar items (e.g., the number of harsh brakings per hour) and some vector items (e.g., percentage of time in each driving scenario type). Other configuration values concern the specification of the tags, that are strings that will be available in the user interface (UI) menus, and the specification of the constraints, that are used for specifying the relationships between tags, in order to support the automatic filling of the UI menus. Once correctly configured, the L3Pilot Measurify installation can be regularly used, i.e., for uploading and downloading the measurements.

#### User Roles and Data Access Rights

Measurify offers the possibility of providing three different roles to users: Providers, Analysts and Admin. Moreover, user rights can be assigned in the configuration, so to have finer control on data access for every single user. For instance, only certain features (not all) could be visible to a certain user. The mapping of user roles and rights in the L3Pilot domain is sketched in Table 3.

Data from the CDB API can be made available to the user through clients such as the Graphical User Interface (GUI), described in the next subsection, the Uploader and third parties tools, such as Postman. In this last case, there is no filter on the clients on the fields of a measurement. This would lead to a confidentiality breach, as the Trip and User IDs should not be visible to the user. Thus, the Measurify APIs have been enriched with the Fieldmask property, settable by the administrator, that allows specifying for each user what fields of a measurement are retrievable (e.g., only values and source device).

### 4.5. Graphical User Interface (GUI)

A web-based Graphical User Interface (GUI) application was created to facilitate access to the data available in the CDB. Although the data is available for download using the Uploader script described in the previous subsection, the user interface provides easier means to access, filter and download the data from a web browser.

#### Implementation Details

The choice of a web-based application in contrast to a native application (Windows/Linux/Mac) is motivated, firstly, by completely removing the burden of installing specific software from the user side, as the application is accessible from any web-browser, including mobile platforms. Secondly, adopting a web-based application allows maintaining a single code base, which would not be generally possible in the case of native applications since they require operating system-specific platforms. Thirdly, web-based development allows one to choose from a wide range of development frameworks, Vue.js [33] being the preferred solution for its API simplicity and flexibility, which enables fast development compared to other solutions such as AngularJS or ReactJS [34].

Architecturally, the GUI is the front-end which renders the data and resources obtained by the CDB RESTful API back-end. This interface was created envisioning a balance between user experience and software modularity. While a very flexible design may allow to easily change the GUI from the API back-end, it limits the user experience as generic forms and fields are required in this paradigm. As a compromise, we designed general Query and Download forms that are dynamically loaded with custom options according to presets in the CDB API. This design choice allows changes to scenario names, PIs and conditions to be transparent to the GUI, increasing modularity and avoiding repetition in the code base.

The GUI is deployed efficiently using Docker containers and a Docker image available in DockerHub. The CDB offers an HTTPS-enabled GUI, which is the main portal for project-wide data access.

### 4.6. Functionalities

The Query functionality is split into Vehicular and Subjective data, each with its respective page. The first is based on five query parameters: Query type (also known as a Performance indicator), Experimental condition, Road type, Driver type and Scenario type. The available options for each query parameter are dynamically loaded from the CDB API and filtered according to constraints indicating possible combinations of the query parameters. All parameters except for the first have a null choice, which ignores filtering records by that parameter.

**Figure 6:**
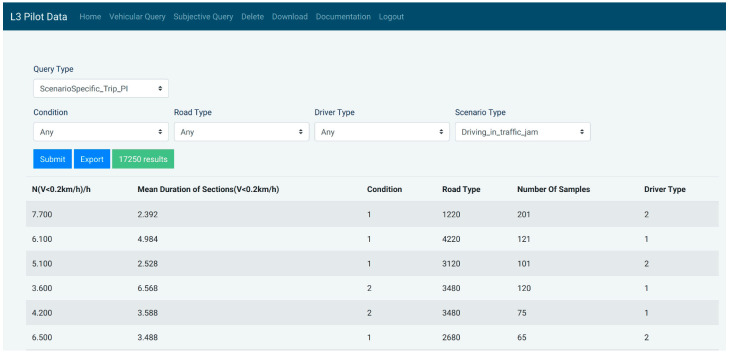
Example of vehicular query with (dummy) results displayed in a table.

Given choices of query parameters, the results are retrieved following a logical AND operation between all the parameter choices, i.e., the records retrieved must match all the query parameter criteria simultaneously. The results are displayed in a table which is populated asynchronously for optimal performance. The table’s column’s titles are obtained dynamically from the CDB, complying with the flexible design thereof. After visualizing the data, the user can also choose to download the resulting records to a CSV file using the Export button. Figure 6 illustrates a query for Scenario Instance PI with different querying parameters.

Similarly, a download page allows users to download all data available in the database by compressing results for all queries into a single file. The results are saved in separate spreadsheets per query type and compressed into a single “.zip”. A progress bar shows which query type is being handled at a given time. This feature has been implemented to allow data analysts to easily take frequent snapshots of all data accessible to their role.

The subjective query page offers only a query type parameter which selects a type of subjective questionnaire from the ones available. The functionality is the same as described for the vehicular query page, despite having only a single query parameter. The solution follows the modular architecture described earlier such that future requirements, such as new query parameters for subjective data, can be easily incorporated into the interface.

The delete functionality is considered in the GUI in case a data owner needs to remove a measurement no longer required (e.g., wrong measurement). The authorized user specifies the trip ID (which is known only to the Provider itself, according to the specified confidentiality rules). 

## 5. Deployment at the Pilot Sites

After lab tests, the CDB was deployed in the cloud, together with the web user interface. In parallel, the Uploader was distributed to all the pilot sites. After the login to the CDB, each data row from the input files is tentatively inserted to the CDB provided that the structure of its data matches the corresponding Feature resource, which is checked for integrity assurance.

In order to make tests on their data and get familiarity with the process, the Pilot sites asked for the possibility of having own instances of the CDB, beside the cloud database, which was reserved for official data, shared among all the vehicle owners and pilot sites, in production. Thus, the Local CDB concept was defined. This was made possible thanks to the open-source release of Measurify. The installation procedure involved the configuration of Atmosphere/Measurify with the latest L3Pilot data structure and the installation of MongoDB, for the actual storage.

Given the complexity of the overall system, some partners asked for simplifying the set-up process. This led to the development of a single Docker containing the Measurify API, with automatic configuration based on Postman scripts, the MongoDB and the GUI accessible through the local host. The image is available on DockerHub.

As the internal organization rules prevented some partners from installing Docker and NodeJS (necessary for running the Uploader) on their own machines, the Information Technology (IT) experts of such organization were asked to install a local server, accessible from different machines in the local network through the web-browser GUI and the Uploader. To avoid installing NodeJS, a stand-alone version of the Uploader was set up. In one pilot site, it was preferred not to make any local installation. To this end, we set up a development installation of the CDB in the cloud.

These different architectural options allowed every partner involved in the pilot sites to get familiarity with the process, according to their different roles. Various patterns of use could be observed. Vehicle owner companies uploaded and checked their data and analysts accessed and analysed data from all the pilot sites but only concerning their specific features.

Needless to say, writing detailed instructions in the project’s collaboration tool was useful to facilitate the usage of the system. Feedback on this from the users helped to improve communication and overall effectiveness, in an iterative process.

Not only did these “early” installations highlight some bugs in the code but they also enabled us to tune the overall process and suggest significant improvements, based on the experience and the analysis of the first sets of actual data. Such suggestions were discussed with the developers and then implemented. Important system functionalities have been added thanks to this collaboration. For instance, we initially considered more experimental conditions than those presented in Section 3. There was also a “Treatment” condition, aggregating a trip’s measurements independent of the status of the ADF—it is sufficient that the ADF is on the vehicle, as opposed to the baseline condition. However, analysts asked to remove this condition, in order to reduce the amount of data to be processed.

A conceptual problem found at the beginning of the deployment was that if the experimental condition changes within an occurring scenario, two scenario instances are created and uploaded to the database although there is only one scenario occurring. This is fine in some scenarios. For others, however, it gives wrong results for, e.g., the duration of a lane change or the standard deviation of the speed during following a lead vehicle. The problem became apparent when looking at lane changes. During the recordings, lane changes are often not performed by the ADF but by the safety driver, or at least signs off on them. In the aggregated data this leads to three scenarios that are uploaded to the database and that are evaluated at the end, which is not the desired output. We thus introduced the concept of “partial” (uninfluenced driving, following a lead vehicle) and “complete” (all the others) scenarios. For partial scenarios, splitting them up, due to an intervening condition change (e.g., from ADF on to ADF off), is fine. For all others, the complete scenario instance is always needed, no matter the condition changes during the scenario. Moreover, all the transitions of conditions that may occur during a complete scenario need to be traced.

Another improvement concerned the maximum length of scenario instances. Since some scenario instances (particularly Uninfluenced driving and Following a lead vehicle) are quite long, in some cases, leading to a low level of meaningfulness for the PIs’ statistical indicators (e.g., min/max/avg/stdev), we were asked to set a maximum length of an instance, for “limited” scenarios. Longer instances of such scenarios are split in fixed-length chunks.

We also implemented the StringMapper tool. All data on the CDB are numeric, as this format was considered best suited for automatic processing of the downloaded data by analysts using their own statistical elaboration packages. An ancillary tool (the StringMapper) was developed on request of some pilot sites, which allows mapping some columns of the downloaded .csv files from codes to the corresponding predefined string values, thus favouring data readability.

As of the end of October 2020, the CDB has been employed in 14 L3Pilot tests sites, from Italy to Sweden, both in its cloud and local versions. Vehicular sensor data are being processed by six impact analysis teams and as many traffic analysis teams, while subjective data by three teams, in order to respond to the research questions.

The cloud CDB, and the GUI as well, are hosted on an Elastic Compute Cloud (EC2) server from Amazon Web Server (AWS). A variety of hardware (CPU, memory, storage) and software (operating system) solutions can be considered, based on the project’s requirements. This solution allows achieving a state-of-the-art level in terms of infrastructure performance, scalability and security. MongoDB also supports sharding, which significantly increases performance by distributing data across multiple machines. Sharding was not necessary in our application case, also because we used PIs, not the raw signal time-series.

At the time of writing, the system has been operational for eight months and the first results have already been achieved by the analysts processing the CDB data. Feedback from analysts and other project partners (vehicle manufacturers and suppliers) is a testament to the ability of the system to meet the requirements. We particularly highlight the ability of extracting and managing the indicators to answer the RQs, while preserving confidentiality, and the workflow requirements (integration with the proprietary workflows and dataflows, recursive CDB upload, .csv download and postediting of the PIs).

Feedback from all the project partners also highlights some good practices that were applied and verified during the project, implementing the workflow depicted in Figure 2:

- an extensive use of abstractions, in order to support functional extensibility and module/code reusability
- the modular approach depicted in Figure 3 for extracting PIs from signal time series revealed itself very useful to deal with a set of specification upgrades, that occurred during the project
- the development of a tool that computes the PIs from the raw data and makes them ready for sharing
- the possibility of postediting the PIs before inserting them in the shared database
- the definition of a tool for efficiently uploading files to the database.
- the development of a web-based, open-source GUI for supporting a proper user experience when querying the database
- the usage of effective, well-established data formats, such as .hdf5, .json, .csv. This was key to guarantee interoperability with different tools, particularly for data logging and data analysis, as research teams are accustomed to various tools, such as VBOX, DL2, Matlab, SPSS, etc.
- the use of state-of-the-art tools for distributed project development (e.g., for code versioning)

A key component of the system is the Measurify measurement-oriented API back-end, which has been appreciated for several reasons, such as:

- Efficient storage and sharing of complex measurements, thanks to the underlying MongoDB nonrelational database management system.
- Easy configurability by specifying the features to be supported in the specific installation (i.e., application database). In the L3Pilot CDB, the features correspond to the types of vehicular and subjective data to be uploaded. Changes in the data structure are easily managed by simply changing the Feature records (old data are to be deleted and reinserted in the new format).
- Ability to seamlessly deal with both vehicular and subjective data
- Open source availability.
- Robustness, as the API was tested in other projects as well.
- Platform-independence, given by the use of the intrinsically platform-independent NodeJS technology and MongoDB open source tool for data storage
- Non-vendor-lockedness. Differently from the typical cloud-based data management solutions, Measurify does not depend on vendor APIs. This makes the service easily portable across cloud providers
- Ease of deployment. The CDB has been deployed in a cloud installation and locally in all the pilot sites, also on laptops.

## 6. Conclusions

This paper has proposed a methodology and implemented a workflow for quantitatively addressing RQs in a collaborative project sharing among partners sensitive big data information (both objective, from vehicular sensors, and subjective, from user questionnaires), which is still not well covered in the literature. We have applied the workflow in L3Pilot, a large-scale project aimed at assessing the impact of automated driving as a safe and efficient means of transportation on public roads. While the project is not finished and its RQs have not been answered yet, the toolchain and the CDB API/GUI have been widely used across all pilot sites. Based on our working experience, we first stress the importance of establishing a collaborative community of researchers and developers who are knowledgeable in their respective domains. This team has been vital to allow a full understanding of the requirements, development of specifications and system and proper handling of all the issues that emerged with the concrete operations in the pilot sites. Discussions between experts in different fields have been very useful to achieve quality in a reasonable timeframe.

The process of sharing data among different providers and analysts for quantitatively answering RQs is complex, as is the development of the supporting systems and tools. Thus, time and effort need to be carefully spent in order to make everything work smoothly. Iterations and flexibility/availability are needed, as specifications (also those established during the project) had to be refined based on the actual test information, also with major design implications.

Our experience has shown the key importance of a reference methodology (in our case, FESTA), to theoretically inform and coherently manage all the steps of the project, in a top-down approach, from higher-level RQs, down to quantitative PIs and the actual signals or questionnaire items needed to answer them. The methodology needs to be put in practice in the everyday work of all the involved research teams, from car manufacturers to data analysts. As we could not find a proper solution ready for the whole chain, we designed a system architecture and developed the missing tools. Particularly, we highlight the following three key components:

- the Common Data Format (CDF) [25], which allowed all partners to deal with all the data in the same format, sharing tools and knowledge, but not the proprietary data, especially those coming from advanced driving assistance systems (ADAS). Not only does the CDF cover the original signal time-series but also additional information (e.g., the driving scenarios), that are computed by the Derived Measures—Performance Indicator Framework.
- the Measurify API, a non-vendor-locked cloud system for sharing appropriate measurements among relevant partners.
- the CDB Aggregator tool, which computes the sharable information, obtained through simple statistical data processing on relevant time segments based on such factors as experimental condition, road type and driving scenario (Figure 3 and Figure 4).

We have described the implementation of the workflow, showing the challenges to overcome to meet the expectations of a real-world SAE automation level 3 pilot project. Feedback from the various types of users of the system (data analysts, original equipment manufacturers and suppliers) has been largely positive and stresses that a proper methodology and a chain of interoperable tools to manage big data are a key factor to the success of a collaborative research project.

While the implementation is exclusively in the automotive field, we argue that the proposed methodological approach and system architecture (Figure 2) and tools are general and could be efficiently adapted and employed in different domains in order to support quantitative research analyses:

- a common data format can be defined for any application domain, if not yet available.
- the Measurify API is released open source and installations can be easily configured for different domains [31], by specifying different features (i.e., measurement types)
- the principles of the CDB Aggregator (segmentation and statistical data synthesis) are generally applicable. Different factors (experimental condition, types of context of usage of a new system to test, etc.) can be efficiently nested in the modular processing schema presented in Figure 3.

The team of automotive and traffic analysts is now processing the data to answer the project RQs, that will be published in other articles. In a longer-term view, the next steps concern the study of more advanced ADFs, also considering the further impact on safety, security, privacy and freedom. Besides this, it will be interesting to apply and verify the proposed workflow and system architecture in other application domains other than automotive.

## Figures and Tables

**Table 1:** An example on definition of logging requirements for a hypothesis [7].

Item	Example
Evaluation area	Technical and traffic
RQ level 1	“What is the impact of the ADF on driving behaviour?”
RQ level 2	“What is the ADF impact on driven speed in different scenarios?”
RQ level 3	“What is the ADF impact on driven speed in driving scenario X?”
Hypothesis	Example 1: “There is no difference in the driven mean speed for the ADF compared to manual driving.” Example 2: “There is no difference in the standard deviation of speed for the ADF compared to manual driving.”
Required Performance indicators (PIs)	Mean speed, standard deviation of speed, max speed, plot (speed/time)
Logging requirements/sensors available	CAN bus of vehicle: Ego speed in x direction

**Table 2:** An overview of the L3Pilot vehicular sensor data performance indicators (PI) types.

PI Type	Description	Example of PIs
Trip PI	PIs computed at trip level	Mean (stdev) longitudinal acceleration, percentage of time elapsed per driving scenario type
Scenario specific Trip PI	PIs computed at trip level but only when a specific driving scenario occurs. Example of driving scenarios, described later, are: driving in a traffic jam, lane change.	Mean duration of sections with speed lower than a threshold
Scenario instance PI	PIs computed for each instance of a driving scenario. The same PIs are computed in each type of scenario	Mean (stdev) time headway, mean(stdev) position in lane
Datapoint for a Following a lead vehicle scenario	Datapoint PIs are computed for each instance of a driving scenario. Different types of scenario have a different datapoint structure. Here we report two examples. Datapoints are used as input for the impact assessment by either resimulating driving scenarios or constructing artificial scenarios based on statistical analyses of scenarios encountered during piloting	Mean (stdev) relative velocity, Time headway at minimum time to collision
Datapoint for Approaching a traffic jam scenario		Vehicle speed at brake or steering onset, Longit. position of object at brake or steering onset

**Table 3:** Measurify user roles and rights. General description and mapping in the L3Pilot case.

Role	Description	L3Pilot Configuration/Notes
Providers	Provider users are data owners. They can upload data and retrieve only their own data.	In L3Pilot, Providers are vehicle owners or their in-depth analysis partner for vehicular sensor data and pilot leaders for subjective data
Analysts	Analyst users cannot upload data but can see all the data of their typology.	In L3Pilot, analysts are the experts responding to the research questions. Utilizing the Measurify’s Right resource, we have implemented three typologies, matching the type of relevant data: Technical and Traffic analysts, that access all vehicular sensor data apart from the Datapoints; Impact analysts, that access Datapoints; User analysts, that access subjective data
Admin	The admin configures the CDB (e.g., setting up the users and rights) and can see (only in case of need) all data entries	Given the adopted ID pseudonymization, the admin cannot resolve IDs (i.e., relating a data entry with its vehicle owner or driver).
